# Dual use and gain-of-function research: a significant endeavor with biosecurity imperatives

**DOI:** 10.1128/msphere.00289-25

**Published:** 2025-06-04

**Authors:** James Giordano, Diane DiEuliis

**Affiliations:** 1 Center for Disruptive Technologies and Future Warfare, Institute of National Strategic Studies, National Defense University 8397, Washington, DC, USA; 2 Center for the Study of Weapons of Mass Destruction, Institute of National Strategic Studies, National Defense University 8397, Washington, DC, USA; University of Michigan, Ann Arbor, Michigan, USA

**Keywords:** biosecurity, gain of function, dual use, COVID, bioweapons

## Abstract

The current U.S. administration has recently proposed a pause on all domestic gain-of-function (GoF) research in order to fully revise existing policy. However, domestic controls on GoF research cannot mandate that other nations follow suit and thus do not prohibit non-compliant nations from engaging in such work. In fact, such national constraints may facilitate opportunities for competitor and adversarial nations (and non-state actors) to advance efforts in this space toward nefarious applications. Moreover, certain groups may argue that GoF research may be necessary for advancing biomedical science (A. Casadevall, F. C. Fang, and M. J. Imperiale, mSphere 9:e00714-23, 2024, https://doi.org/10.1128/msphere.00714-23) and global health security and, through this stance, conduct GoF research that has direct dual-use viability. In this light, we argue that all GoF research should be conducted under a robust framework of enhanced BSL controls that explicitly define its dual usability, classify any such enterprise as DURC, engage regulatory oversight, and establish ethical responsibility within the scope and tenor of international law. This essay describes the possible burdens and risks of GoF research, and in addressing the challenges posed by such work, proposes recommendations for future policy toward sustaining beneficial outcomes and preventing or mitigating threats to public health and global biosecurity.

## INTRODUCTION

The current U.S. administration has recently proposed a pause on all domestic gain-of-function (GoF) research (1). This is notable, given that the origins of SARS-CoV-2, the causative viral strain of COVID-19, have been controversial since the start of the pandemic in early 2020. Initially proposed as a naturally occurring zoonosis, the fact that the Wuhan Institute of Virology (WIV) was working with engineered strains of SARS isolated from bats prompted theories that the virus leaked from the WIV through accidental release or was intentionally modified to be a biological weapon. As we have noted (2), extant available evidence to date does not appear to indicate (i) that the SARS-CoV-2 virus was an intentionally developed biological weapon and/or (ii) that the release of the SARS-CoV-2 virus or equivalently pathogenic pre- or co-cursors was intentional.

However, we have previously opined that given ongoing dual use research of concern (DURC), and specifically gain-of-function (GoF) research engaged at the WIV, it was indeed possible—and we argued highly likely—that some viral precursor may have been inadvertently transported from the laboratory into a native environment, either via a laboratory-acquired infection or other inadvertent release (1). This has been corroborated by recent reports (3) and the current perspective offered by the U.S. (and German) intelligence community (https://justthenews.com/government/security/german-spy-agency-convinced-covid-19-likely-came-wuhan-lab; https://www.cbsnews.com/news/cia-covid-likely-originated-lab-low-confidence-assessment/). It is possible that the pathogenicity and/or zoonicity of such viral products were not estimated to pose a risk or threat to humans by researchers at the WIV, and thus the biosecurity level (BSL) under which these stages of GoF research were conducted and maintained may have been low. But despite the BSL level employed, evidence suggests that the WIV itself was not up to stringent standards of biosafety and biosecurity (4). As GoF research is intended to and often successfully does incur structural modifications to viral genomes capable of affecting their activity, it is highly probable that such a modified viral product was more labile than recognized and once released into native ecologies rapidly transformed into an entity with robust transmission to intermediate species and ultimately zoonotic infective capability.

In light of such concerns over biosecurity risks and the potential for laboratory-associated outbreaks, the recent posture of the current U.S. administration reflects broader consideration of DURC and GoF research as the focus of intense scientific and ethical debate (5). However, domestic bans on GoF research do not necessarily prohibit such work from being conducted in other nations, and, in fact, may facilitate opportunities for competitor and adversarial nations (and non-state actors) to advance efforts in this space toward nefarious applications. Therefore, we argue that any continuation of such research must be contingent upon stringent enhancements to biosecurity and biosafety measures being applied to ensure that the benefits do not come at the cost of unnecessary risk to public health.

We realize that certain groups may argue that GoF research may be necessary for advancing biomedical science (1) and global health security. While this may be a defensible stance, we counter that in all cases, GoF research must be conducted under a robust framework of enhanced BSL controls that explicitly define its dual usability, classify any such enterprise as DURC, engage regulatory oversight, and establish ethical responsibility within the scope and tenor of international law.

## SCIENTIFIC RATIONALE FOR GAIN-OF-FUNCTION RESEARCH

Gain-of-function research refers to the deliberate modification of an organism—typically a pathogen—to alter/enhance certain characteristics, such as transmissibility, virulence, or host range (6). The objective of such research is to anticipate and prepare for naturally occurring mutations that could lead to more infectious and dangerous strains of infectious agents. One of the primary justifications for GoF research is its ability to provide insight into the mechanisms by which pathogens evolve (7). By inducing and studying specific genetic changes, factors that contribute to zoonotic spillover, viral adaptation, and drug resistance can be elucidated. Such knowledge is essential for (i) forecasting patterns of disease spread, epidemic viability, and potential pandemics and (ii) developing proactive interventional tactics and strategies (e.g., disease prevention or mitigation).

For example, studies on highly pathogenic influenza strains, such as H5N1 and H7N9, have demonstrated how relatively minor genetic modifications can significantly alter host specificity and transmission dynamics. Similarly, research into coronaviruses—including SARS-CoV and MERS-CoV—has been instrumental in understanding receptor binding properties, immune evasion mechanisms, and the likelihood of animal-to-human (i.e., zoonotic) and human-to-human transmission. Clearly, these types of studies fortify abilities to predict and counteract emerging infectious threats worldwide.

Thus, GoF research can engage a crucial role in biomedical countermeasure development. By understanding the pathways through which viruses acquire resistance to antiviral drugs, more robust diagnostics and therapeutics can be preemptively designed. This approach has been employed in the study of influenza, HIV, and coronaviruses to identify vulnerabilities that can be exploited for vaccine and drug development. Moreover, by engineering attenuated but immunogenic strains of viruses, GoF research contributes directly to vaccine innovation (8). This has been exemplified in the development of live-attenuated vaccines, which rely on a controlled modulation of virulence to stimulate immune responses without causing disease.

## ENHANCING BIOSECURITY AND PREPAREDNESS

Simply put, we believe that GoF research—if conducted responsibly—can improve global health security. By characterizing potential epidemic and pandemic threats before they emerge naturally, public health agencies can develop and stockpile countermeasures, refine diagnostic tools, and implement targeted surveillance systems. This proactive approach is fundamentally superior to reactive crisis management, as evidenced by the delays in developing effective diagnostics and vaccines during the early stages of the COVID-19 pandemic (9).

Yet, while the benefits of GoF research are substantial, its risks cannot—and should not—be ignored. The primary concerns associated with this work include laboratory biosafety failures and the potential for misuse by state or non-state actors. One of the most immediate risks of GoF research is the possibility of accidental pathogen release. History has shown that even high-containment laboratories that are not performing GoF research are not immune to breaches. In addition to recent implications that SARS-CoV-2 originated in and was unintentionally released from a laboratory (see above), prior examples of such mishaps include earlier accidental releases in Asia (10, 11), as well as the accidental release of smallpox from a UK laboratory in 1978 (12); and the escape of anthrax spores from a Soviet facility in 1979 (13).

Although such events have historically been infrequent, ongoing developments in gene editing and synthetic biological techniques and tools highlight the fact that conducting GoF research should only be performed in laboratories that adhere to the highest biosafety and biosecurity standards. But we further emphasize that the capabilities conferred by these new methods and tools—taken with the potential for unintentional exposure or environmental release—prompt reconsideration of existing criteria for BSL assignment and underscore the need for more stringent regulatory frameworks and oversight.

## REGARDING DUAL USE

The dual-use nature of GoF research (viz., that scientific findings intended for public health benefits could be misappropriated for nefarious purposes) extends concern beyond bioterrorism to include geopolitical conflicts in which state actors may seek to develop offensive biological capabilities under the guise of legitimate research. To wit, when GoF research is applied to infectious pathogens or has the ability to create harmful biological entities, it is of high concern. In 2017, the United States revised its initial GoF policy into a policy for potential pandemic pathogen care and oversight (P3CO) (14). Its goal was to prevent misuse of GoF that might cause wide-scale human pandemics and/or human harm.

However, in light of the tools of emerging biotechnology and a burgeoning global bioeconomy, there are now wider swaths of DURC and/or GoF that could have harmful biological outcomes in addition to pandemics, to include harm to agriculture or the environment, societal instability, or other harmful bioincidents.

The revelation that certain pathogens can be engineered for increased transmissibility or virulence presents an inherent security dilemma. On the one hand, transparency in research is critical for scientific progress—and public safety and health; but on the other, unrestricted dissemination of GoF findings risks enabling peer competitors and adversaries to exploit these advances for nefarious purposes. The current reevaluation of P3CO provides an opportunity to address these issues.

## THE NEED FOR INCREASED STRINGENCY IN BIOSECURITY

To enable navigating this dilemma, and given the risks associated with GoF research, we argue that its continuation must be accompanied by a commensurate strengthening of biosafety and biosecurity protocols (15, 16). In advancing such a posture, we propose that any genuine effort(s) toward these ends must entail the following.

### Biosecurity by design

We have advocated for “biosecurity by design” in this context (17) whereby, regardless of whether a DURC or GoF experimentation involves human pathogens or agents on the select agent list, the experimentation should be evaluated for risks before any kinetic research is performed. This provides that intentional research is screened to ensure that GoF (or other dual-use capability) is the only option to achieve a critical public health goal. Other means should be explored to ascertain that there are no other viable ways of answering the same question (for example, through the use of less pathogenic microbes [18], truncated or minimal cellular or viral models as proxies, or even exploration of the question using *in silico* models). Indeed, while we applaud the existing DURC and PC3O frameworks, we concur with the most recent NSABB report that noted significant overlap between DURC and PC3O and the dangers of too narrowly defining research in these categories. An approach using biosecurity by design, if applied more broadly and routinely, can mitigate a broader scope of risk, particularly in scenarios in which risk has not been previously evaluated.

### Elevating biosecurity levels for both high-risk pathogens and high/rapid translational methods

BSL-3+ and BSL-4 laboratories must become the standard for any GoF research involving pathogens with pandemic potential. These facilities should be subject to rigorous certification, routine audits, and real-time monitoring to ensure compliance with international biosafety standards. Moreover, researchers conducting such work should undergo continuous training in biocontainment practices.

### Implementing a global oversight framework

A centralized global oversight body—potentially under the auspices of the World Health Organization (WHO) or a dedicated United Nations (UN) agency—should regulate and monitor GoF research. This body would establish uniform guidelines, assess risk-benefit analyses, and facilitate information sharing among nations while preventing the unregulated proliferation of sensitive research.

### Enhancing transparency and ethical review

The ethical implications of GoF research are profound. If not conducted under rigorous oversight, it raises questions about the moral responsibility of scientists in balancing risk and benefit. Furthermore, public perception of this work has become increasingly negative, particularly in the wake of the COVID-19 pandemic. All GoF research proposals should undergo mandatory ethical review, evaluating both scientific merit and potential societal risks. Additionally, an international registry of GoF studies should be maintained to ensure accountability and enable rapid risk assessment should concerns arise. Rebuilding trust in biomedical research requires a commitment to transparency, accountability, and enhanced safety measures.

### Strengthening biosecurity measures against misuse

To mitigate the risk of GoF research being exploited for malicious purposes, strict biosecurity protocols must be in place. This includes controlled access to high-risk laboratories, enhanced screening of personnel, and the development of forensic monitoring tools to track and prevent unauthorized use of GoF methodologies.

With regard to monitoring tools, this requires the ability to understand disease emergence through better epidemiological technologies, forensics capabilities, along with the ability to attribute disease emergence. While some assert that SARS-CoV-2 origins are less important than achieving a robust response, we respectfully disagree—the ability to forensically identify and attribute the emergence of a biological entity, whether naturally occurring, accidental, or purposeful, stands as a significant and essential tool for deterring biothreats; moreover, our inability to achieve this at the start of COVID-19 stands as a clear vulnerability that should be addressed.

### Improving public communication and risk education

Scientists and policymakers must engage in transparent dialogue with the public about the necessity of GoF research, the precautions in place, and the measures taken to mitigate associated risks. This will be critical for maintaining public trust and ensuring informed discourse on the ethical dimensions of this work.

Toward these ends, we offer this set of defined, discrete recommendations.

- All GoF research must define dual usability upon proposal/outset.
- All GoF research must define and list in an internationally transparent data repository the (biological) outcomes and products of each phase of effort.
- All GoF research must be conducted under at least BSL2, regardless of the phase of effort.
- GoF efforts—and outcomes/products—should be monitored by an (internationally viable and valid) external risk assessment entity to assess whether the BSL should be increased in light of perceived escalation of public health and/or dual use burden or threat-of-harm (to humans and/or their ecology).

## CONCLUSION

We assert that GoF research remains an valuable tool against infectious diseases, providing critical insights into pathogen evolution, pandemic preparedness, and vaccine development. However, its continuation is justifiable only if accompanied by an equally rigorous commitment to enhancing biosecurity measures. Strengthening biosafety protocols, establishing robust regulatory oversight, and fostering ethical responsibility are imperative to ensure that the benefits of this research outweigh its risks. Rather than banning GoF research outright—a reactionary stance that would impede scientific progress—we argue that the global scientific community must instead focus on creating a framework that maximizes safety while preserving the transformative potential of this research. In doing so, both scientific integrity and its ethical probity and thus the value to safeguard public health and global security are preserved.
